# *trans*-Chlorido(phenanthren-9-yl)bis­(triphenyl­phosphane)nickel(II)

**DOI:** 10.1107/S1600536811032326

**Published:** 2011-08-27

**Authors:** Xiangyang Lei, Karla A. Obregon

**Affiliations:** a Department of Chemistry & Biochemistry, Lamar University, Beaumont, TX 77710, USA

## Abstract

The title compound, [Ni(C_14_H_9_)Cl(C_18_H_15_P)_2_], was synthesized from the reaction between 9-chloro­phenanthrene, NiCl_2_·6H_2_O and triphenyl­phosphane in ethanol. The bond angles around the Ni^II^ atom indicate that it exists in a slightly distorted square-planar geometry.

## Related literature

For the synthesis, see: Soolinger *et al.* (1990 ▶). For analogues and related applications, see: Rosen *et al.* (2011 ▶); Zim *et al.* (2001 ▶); Chen & Yang (2007*a* ▶,*b* ▶); Gao & Yang (2008 ▶); Zhou *et al.* (2009 ▶); Roma *et al.* (2011 ▶); Liu *et al.* (2008 ▶).
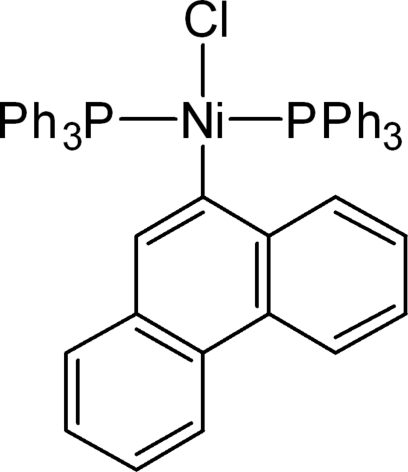


## Experimental

### 

#### Crystal data

[Ni(C_14_H_9_)Cl(C_18_H_15_P)_2_]
*M* *_r_* = 795.91
Orthorhombic, 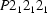

*a* = 11.090 (5) Å
*b* = 15.204 (7) Å
*c* = 23.679 (10) Å
*V* = 3993 (3) Å^3^
*Z* = 4
Mo *K*α radiation
μ = 0.67 mm^−1^
*T* = 110 K
0.59 × 0.46 × 0.23 mm

#### Data collection

Bruker APEXII CCD diffractometer
Absorption correction: multi-scan (*SADABS*; Bruker, 2008 ▶) *T*_min_ = 0.692, *T*_max_ = 0.863
53371 measured reflections
6780 independent reflections
6359 reflections with *I* > 2σ(*I*)
*R*_int_ = 0.048

#### Refinement

*R*[*F* ^2^ > 2σ(*F* ^2^)] = 0.024
*wR*(*F* ^2^) = 0.057
*S* = 1.02
6780 reflections
488 parameters
H-atom parameters constrained
Δρ_max_ = 0.26 e Å^−3^
Δρ_min_ = −0.14 e Å^−3^
Absolute structure: Flack (1983 ▶), 2986 Friedel pairs
Flack parameter: 0.000 (7)

### 

Data collection: *APEX2* (Bruker, 2004 ▶); cell refinement: *SAINT* (Bruker, 2004 ▶); data reduction: *SAINT*; program(s) used to solve structure: *SHELXS97* (Sheldrick, 2008 ▶); program(s) used to refine structure: *SHELXL97* (Sheldrick, 2008 ▶); molecular graphics: *SHELXTL* (Sheldrick, 2008 ▶); software used to prepare material for publication: *SHELXTL*.

## Supplementary Material

Crystal structure: contains datablock(s) I, global. DOI: 10.1107/S1600536811032326/vm2114sup1.cif

Structure factors: contains datablock(s) I. DOI: 10.1107/S1600536811032326/vm2114Isup2.hkl

Supplementary material file. DOI: 10.1107/S1600536811032326/vm2114Isup3.cdx

Additional supplementary materials:  crystallographic information; 3D view; checkCIF report

## Figures and Tables

**Fig. 1.**
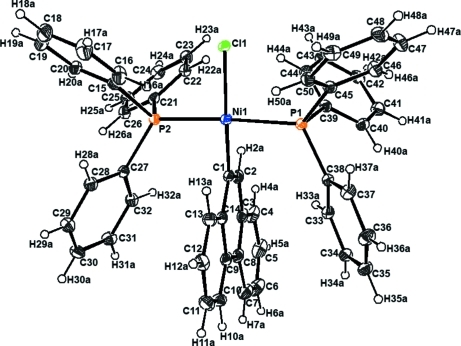
A view of the molecular structure. Ellipsoids are drawn at the 30% probability level.

## supplementary crystallographic information

### Comment

Ni catalysts have attracted considerate attention in recent years. In comparison with Pd and Pt catalysts, Ni catalysts are more desirable from the standpoints of economics and versatility (Rosen *et al.*, 2011). Ni-catalyzed cross-coupling reactions play an important role in the formation of carbon-carbon and carbon-heteroatom bonds. The mechanism of Ni-catalyzed cross-coupling reactions was considered similar to the Pd-catalyzed cross-couplings (Rosen *et al.*, 2011). The catalytic cycle in both cases involves three sequential steps: oxidative addition, translation, and reductive elimination (Zim *et al.*, 2001). Ni(II) σ-aryl complex is believed to be the oxidative addition product in the Ni-catalyzed cross-coupling reactions. Soolinger *et al.* (1990), Chen & Yang (2007*a*,*b*), and Gao & Yang (2008) have demonstrated that isolatable Ni(II) σ-aryl complexes can be directly used as efficient catalysts for cross-coupling reactions. In addition, Zhou *et al.* (2009) have reported that Ni(II) σ-aryl complexes can catalyze dehalogenation of aryl chlorides, and Roma *et al.* (2011)have shown that Ni(II) σ-aryl complexes can promote the polymerization of methylmethacrylate.

As further advances in Ni(II) σ-aryl complexes as catalyst are necessary, we synthesized the title compound in an analogous fashion to the literature procedure (Soolinger *et al.*, 1990). The title compound is air- and thermally stable. The bond angles around Ni of the complex indicate that it exists in a slightly distorted square-planar geometry, which is similar to the geometry of its 1-naphthyl (Zhou *et al.*, 2009) and 4-acetylnaphthyl (Liu *et al.*, 2008) analogues. It is noteworthy that there are potentially C—H···Cl hydrogen bond intramolecular interactions, and the donor-acceptor distances are 2.862 Å for C22—H22A···Cl1 and 2.887 Å for C44—H44A···Cl1. There are also potentially C—H···Cl intermolecular interactions, and the donor-acceptor distances are 2.747 Å for C23—H23A···Cl1^i^ and 2.872 Å for C43—H43A···Cl1^i^ (symmetry codes: (i) -1/2 + *x*, 3/2 - *y*, - *z*). Intramolecular C—H···π interactions are observed as the distances from C32—H32A and C33—H33A to the centroid of the plane C1—C2—C3—C8—C9—C14 of the phenanthrene ring are 2.788 and 2.588 Å, respectively. The application of the title compound as catalyst in cross-coupling reactions is under investigation.

### Experimental

A stirred mixture of 1.20 g (5.0 mmol) of NiCl_2_.6H_2_O, 2.88 g (11.0 mmol) of triphenylphosphine and 25 ml of 96% ethanol was heated until a gentle reflux started. 9-Chlorophenanthrene (10.0 mmol, 2.13 g, excess) was then added, followed by zinc dust (0.33 g, 5.0 mmol, Merck, analytical grade) over 5 min. The dark-green mixture very soon turned yellow. After stirring and heating under reflux for 1.5 h (under nitrogen), the mixture was cooled to 293 K. Four 2 ml portions of 30% aqueous hydrochloric acid were added over 15 min. After stirring for 1.5 h, the solid was filtered off on a sintered-glass funnel and successively washed with 5 ml of ethanol, twice with 5 ml of 1 *M* aqueous hydrochloric acid, twice with 5 ml of 96% ethanol and once with 5 ml of pentane. The yellowish solid (3.10 g) was dried *in vacuo*. Single crystals suitable for X-ray diffraction were obtained by recrystallization from CH_2_Cl_2_/hexanes.

### Refinement

All non-hydrogen atoms were refined with anisotropic thermal parameters. The hydrogen atoms bound to carbon atoms were placed in idealized positions and constrained to ride on their parent atoms, with d(C–H) = 0.95 Å, *U*_iso_(H) = 1.2*U*_eq_(C).

### Crystal data

**Table:**

[Ni(C_14_H_9_)Cl(C_18_H_15_P)_2_]	*F*(000) = 1656
*M**_r_* = 795.91	*D*_x_ = 1.324 Mg m^−^^3^
Orthorhombic, *P*2_1_2_1_2_1_	Mo *K*α radiation, λ = 0.71073 Å
Hall symbol: P 2ac 2ab	Cell parameters from 6845 reflections
*a* = 11.090 (5) Å	θ = 2.2–26.7°
*b* = 15.204 (7) Å	µ = 0.67 mm^−^^1^
*c* = 23.679 (10) Å	*T* = 110 K
*V* = 3993 (3) Å^3^	Plate, orange
*Z* = 4	0.59 × 0.46 × 0.23 mm

### Data collection

**Table:**

Bruker APEXII CCD diffractometer	6780 independent reflections
Radiation source: fine-focus sealed tube	6359 reflections with *I* > 2σ(*I*)
graphite	*R*_int_ = 0.048
φ and ω scans	θ_max_ = 25.0°, θ_min_ = 2.0°
Absorption correction: multi-scan (*SADABS*; Bruker, 2008)	*h* = −13→13
*T*_min_ = 0.692, *T*_max_ = 0.863	*k* = −18→18
53371 measured reflections	*l* = −28→28

### Refinement

**Table:**

Refinement on *F*^2^	Secondary atom site location: difference Fourier map
Least-squares matrix: full	Hydrogen site location: inferred from neighbouring sites
*R*[*F*^2^ > 2σ(*F*^2^)] = 0.024	H-atom parameters constrained
*wR*(*F*^2^) = 0.057	*w* = 1/[σ^2^(*F*_o_^2^) + (0.0354*P*)^2^] where *P* = (*F*_o_^2^ + 2*F*_c_^2^)/3
*S* = 1.02	(Δ/σ)_max_ = 0.002
6780 reflections	Δρ_max_ = 0.26 e Å^−^^3^
488 parameters	Δρ_min_ = −0.14 e Å^−^^3^
0 restraints	Absolute structure: Flack (1983), 2986 Friedel pairs
Primary atom site location: structure-invariant direct methods	Flack parameter: 0.000 (7)

### Special details

**Table:**

Geometry. All e.s.d.'s (except the e.s.d. in the dihedral angle between two l.s. planes) are estimated using the full covariance matrix. The cell e.s.d.'s are taken into account individually in the estimation of e.s.d.'s in distances, angles and torsion angles; correlations between e.s.d.'s in cell parameters are only used when they are defined by crystal symmetry. An approximate (isotropic) treatment of cell e.s.d.'s is used for estimating e.s.d.'s involving l.s. planes.
Refinement. Refinement of *F*^2^ against ALL reflections. The weighted *R*-factor *wR* and goodness of fit *S* are based on *F*^2^, conventional *R*-factors *R* are based on *F*, with *F* set to zero for negative *F*^2^. The threshold expression of *F*^2^ > σ(*F*^2^) is used only for calculating *R*-factors(gt) *etc.* and is not relevant to the choice of reflections for refinement. *R*-factors based on *F*^2^ are statistically about twice as large as those based on *F*, and *R*-factors based on ALL data will be even larger.

### Fractional atomic coordinates and isotropic or equivalent isotropic displacement parameters (Å^2^)

**Table:**

	*x*	*y*	*z*	*U*_iso_*/*U*_eq_	
Ni1	0.56721 (2)	0.527489 (15)	−0.047248 (9)	0.02000 (7)	
Cl1	0.49242 (4)	0.63193 (3)	0.009025 (18)	0.02449 (11)	
P1	0.65475 (5)	0.45655 (3)	0.024960 (19)	0.02055 (11)	
P2	0.50725 (4)	0.59607 (3)	−0.125640 (19)	0.02011 (11)	
C1	0.65053 (18)	0.44916 (12)	−0.09623 (8)	0.0215 (4)	
C2	0.76625 (18)	0.46946 (15)	−0.11185 (8)	0.0264 (4)	
H2A	0.8004	0.5235	−0.0997	0.032*	
C3	0.83753 (19)	0.41080 (15)	−0.14631 (8)	0.0285 (5)	
C4	0.95831 (19)	0.43137 (16)	−0.15978 (10)	0.0373 (6)	
H4A	0.9910	0.4862	−0.1482	0.045*	
C5	1.0289 (2)	0.37379 (18)	−0.18910 (11)	0.0481 (7)	
H5A	1.1103	0.3884	−0.1974	0.058*	
C6	0.9811 (3)	0.29272 (18)	−0.20702 (11)	0.0503 (7)	
H6A	1.0305	0.2524	−0.2272	0.060*	
C7	0.8645 (2)	0.27199 (17)	−0.19557 (9)	0.0409 (6)	
H7A	0.8331	0.2174	−0.2083	0.049*	
C8	0.7884 (2)	0.33008 (14)	−0.16497 (9)	0.0298 (5)	
C9	0.66463 (19)	0.30797 (14)	−0.15135 (8)	0.0272 (5)	
C10	0.6079 (2)	0.23092 (15)	−0.16979 (9)	0.0355 (5)	
H10A	0.6516	0.1905	−0.1925	0.043*	
C11	0.4903 (2)	0.21231 (15)	−0.15580 (9)	0.0387 (6)	
H11A	0.4536	0.1598	−0.1691	0.046*	
C12	0.4251 (2)	0.26995 (14)	−0.12229 (9)	0.0343 (5)	
H12A	0.3441	0.2567	−0.1124	0.041*	
C13	0.47822 (19)	0.34736 (14)	−0.10304 (8)	0.0292 (5)	
H13A	0.4328	0.3866	−0.0802	0.035*	
C14	0.59785 (18)	0.36821 (13)	−0.11689 (8)	0.0242 (4)	
C15	0.36705 (18)	0.65927 (14)	−0.12249 (8)	0.0253 (4)	
C16	0.2761 (2)	0.63172 (17)	−0.08626 (9)	0.0359 (5)	
H16A	0.2892	0.5835	−0.0615	0.043*	
C17	0.1658 (2)	0.67511 (19)	−0.08639 (10)	0.0473 (7)	
H17A	0.1035	0.6565	−0.0616	0.057*	
C18	0.1462 (2)	0.74547 (18)	−0.12253 (10)	0.0451 (6)	
H18A	0.0710	0.7753	−0.1221	0.054*	
C19	0.2356 (2)	0.77222 (16)	−0.15896 (10)	0.0379 (6)	
H19A	0.2215	0.8199	−0.1840	0.046*	
C20	0.34575 (19)	0.72989 (14)	−0.15910 (9)	0.0286 (5)	
H20A	0.4074	0.7488	−0.1842	0.034*	
C21	0.62413 (17)	0.67544 (13)	−0.14485 (8)	0.0230 (4)	
C22	0.69398 (18)	0.71257 (14)	−0.10193 (9)	0.0259 (5)	
H22A	0.6773	0.6984	−0.0637	0.031*	
C23	0.7876 (2)	0.77001 (15)	−0.11455 (9)	0.0315 (5)	
H23A	0.8341	0.7953	−0.0850	0.038*	
C24	0.81292 (19)	0.79046 (15)	−0.17089 (9)	0.0318 (5)	
H24A	0.8782	0.8285	−0.1797	0.038*	
C25	0.74276 (19)	0.75518 (14)	−0.21397 (10)	0.0331 (5)	
H25A	0.7591	0.7699	−0.2522	0.040*	
C26	0.64865 (19)	0.69838 (14)	−0.20099 (8)	0.0265 (5)	
H26A	0.6004	0.6748	−0.2305	0.032*	
C27	0.48354 (17)	0.53297 (13)	−0.19152 (7)	0.0222 (4)	
C28	0.36738 (19)	0.51242 (14)	−0.20905 (8)	0.0278 (5)	
H28A	0.3002	0.5325	−0.1878	0.033*	
C29	0.3486 (2)	0.46246 (15)	−0.25774 (9)	0.0349 (5)	
H29A	0.2687	0.4483	−0.2690	0.042*	
C30	0.4445 (2)	0.43348 (15)	−0.28973 (9)	0.0353 (5)	
H30A	0.4310	0.4001	−0.3231	0.042*	
C31	0.5605 (2)	0.45350 (14)	−0.27274 (8)	0.0312 (5)	
H31A	0.6272	0.4337	−0.2945	0.037*	
C32	0.58031 (19)	0.50237 (13)	−0.22399 (8)	0.0258 (4)	
H32A	0.6605	0.5152	−0.2126	0.031*	
C33	0.76549 (19)	0.30173 (14)	−0.01596 (8)	0.0267 (5)	
H33A	0.8154	0.3402	−0.0372	0.032*	
C34	0.7797 (2)	0.21147 (14)	−0.02159 (9)	0.0294 (5)	
H34A	0.8389	0.1888	−0.0467	0.035*	
C35	0.7080 (2)	0.15427 (15)	0.00917 (9)	0.0336 (5)	
H35A	0.7178	0.0925	0.0052	0.040*	
C36	0.6218 (2)	0.18761 (14)	0.04567 (11)	0.0357 (5)	
H36A	0.5718	0.1486	0.0666	0.043*	
C37	0.60811 (18)	0.27826 (13)	0.05175 (9)	0.0296 (5)	
H37A	0.5499	0.3006	0.0775	0.036*	
C38	0.67883 (17)	0.33683 (13)	0.02052 (8)	0.0222 (4)	
C39	0.80445 (17)	0.50331 (13)	0.03660 (7)	0.0223 (4)	
C40	0.90278 (18)	0.45309 (13)	0.05382 (8)	0.0275 (5)	
H40A	0.8941	0.3915	0.0591	0.033*	
C41	1.0134 (2)	0.49305 (15)	0.06328 (9)	0.0315 (5)	
H41A	1.0800	0.4588	0.0756	0.038*	
C42	1.02723 (19)	0.58275 (15)	0.05485 (9)	0.0325 (5)	
H42A	1.1038	0.6094	0.0604	0.039*	
C43	0.9301 (2)	0.63360 (13)	0.03836 (8)	0.0320 (5)	
H43A	0.9395	0.6951	0.0329	0.038*	
C44	0.81898 (18)	0.59436 (14)	0.02980 (8)	0.0265 (4)	
H44A	0.7518	0.6295	0.0192	0.032*	
C45	0.58119 (18)	0.46590 (13)	0.09426 (7)	0.0235 (4)	
C46	0.6465 (2)	0.46476 (15)	0.14471 (8)	0.0316 (5)	
H46A	0.7321	0.4650	0.1435	0.038*	
C47	0.5875 (2)	0.46324 (17)	0.19689 (8)	0.0380 (5)	
H47A	0.6328	0.4635	0.2310	0.046*	
C48	0.4638 (2)	0.46144 (15)	0.19862 (8)	0.0345 (5)	
H48A	0.4233	0.4591	0.2339	0.041*	
C49	0.3976 (2)	0.46299 (15)	0.14867 (9)	0.0311 (5)	
H49A	0.3120	0.4622	0.1500	0.037*	
C50	0.45626 (18)	0.46568 (14)	0.09676 (8)	0.0264 (4)	
H50A	0.4105	0.4674	0.0629	0.032*	

### Atomic displacement parameters (Å^2^)

**Table:**

	*U*^11^	*U*^22^	*U*^33^	*U*^12^	*U*^13^	*U*^23^
Ni1	0.02228 (12)	0.01963 (13)	0.01808 (11)	0.00345 (10)	0.00132 (10)	−0.00025 (10)
Cl1	0.0339 (3)	0.0210 (2)	0.0185 (2)	0.0106 (2)	0.00216 (19)	−0.00120 (17)
P1	0.0232 (2)	0.0196 (3)	0.0189 (2)	0.0014 (2)	0.00066 (19)	0.00014 (19)
P2	0.0200 (2)	0.0211 (3)	0.0192 (2)	0.0012 (2)	−0.0001 (2)	0.00030 (19)
C1	0.0253 (10)	0.0201 (11)	0.0192 (9)	0.0044 (8)	−0.0008 (8)	0.0023 (8)
C2	0.0291 (11)	0.0271 (11)	0.0230 (10)	0.0051 (10)	0.0002 (8)	0.0050 (9)
C3	0.0296 (11)	0.0336 (12)	0.0224 (10)	0.0084 (10)	0.0051 (9)	0.0095 (9)
C4	0.0291 (12)	0.0431 (14)	0.0397 (12)	0.0084 (10)	0.0064 (10)	0.0168 (11)
C5	0.0366 (14)	0.0590 (18)	0.0486 (14)	0.0147 (12)	0.0174 (11)	0.0199 (13)
C6	0.0603 (17)	0.0449 (16)	0.0458 (14)	0.0219 (14)	0.0245 (13)	0.0119 (12)
C7	0.0564 (17)	0.0369 (14)	0.0294 (12)	0.0162 (12)	0.0137 (11)	0.0041 (10)
C8	0.0387 (12)	0.0292 (12)	0.0214 (10)	0.0093 (10)	0.0030 (9)	0.0065 (9)
C9	0.0369 (12)	0.0262 (11)	0.0186 (10)	0.0055 (10)	0.0016 (9)	0.0051 (8)
C10	0.0581 (16)	0.0252 (12)	0.0231 (11)	0.0030 (11)	0.0017 (10)	−0.0013 (9)
C11	0.0580 (16)	0.0295 (12)	0.0286 (11)	−0.0104 (12)	−0.0067 (11)	0.0028 (9)
C12	0.0386 (13)	0.0324 (12)	0.0317 (11)	−0.0112 (11)	−0.0052 (10)	0.0069 (10)
C13	0.0368 (12)	0.0267 (11)	0.0242 (10)	0.0001 (9)	0.0009 (9)	0.0027 (8)
C14	0.0317 (11)	0.0206 (10)	0.0204 (9)	0.0016 (9)	−0.0016 (8)	0.0041 (8)
C15	0.0226 (10)	0.0305 (12)	0.0229 (10)	0.0022 (9)	−0.0035 (8)	−0.0069 (9)
C16	0.0275 (12)	0.0529 (15)	0.0273 (11)	0.0050 (11)	0.0031 (9)	0.0064 (10)
C17	0.0261 (12)	0.081 (2)	0.0345 (12)	0.0106 (13)	0.0081 (10)	0.0030 (13)
C18	0.0279 (13)	0.0627 (18)	0.0446 (14)	0.0183 (12)	−0.0054 (11)	−0.0085 (13)
C19	0.0352 (13)	0.0349 (13)	0.0437 (14)	0.0099 (11)	−0.0099 (11)	−0.0031 (11)
C20	0.0248 (11)	0.0279 (12)	0.0330 (11)	0.0030 (9)	−0.0052 (9)	−0.0019 (9)
C21	0.0202 (10)	0.0205 (11)	0.0285 (10)	0.0029 (8)	−0.0024 (8)	0.0030 (8)
C22	0.0271 (11)	0.0264 (11)	0.0241 (10)	0.0014 (9)	−0.0014 (9)	0.0011 (8)
C23	0.0269 (11)	0.0310 (12)	0.0365 (12)	−0.0009 (10)	−0.0075 (9)	−0.0010 (10)
C24	0.0244 (11)	0.0301 (12)	0.0410 (13)	−0.0030 (9)	0.0033 (10)	0.0042 (10)
C25	0.0323 (13)	0.0340 (13)	0.0329 (12)	−0.0025 (10)	0.0030 (10)	0.0056 (10)
C26	0.0264 (11)	0.0278 (11)	0.0254 (10)	−0.0023 (9)	−0.0023 (9)	0.0032 (9)
C27	0.0260 (10)	0.0198 (10)	0.0208 (8)	0.0011 (9)	−0.0009 (8)	0.0023 (8)
C28	0.0267 (11)	0.0272 (12)	0.0295 (10)	0.0003 (9)	−0.0012 (9)	−0.0008 (9)
C29	0.0354 (12)	0.0337 (12)	0.0357 (11)	−0.0023 (11)	−0.0091 (10)	−0.0031 (10)
C30	0.0488 (14)	0.0311 (12)	0.0260 (10)	−0.0010 (11)	−0.0045 (11)	−0.0048 (9)
C31	0.0388 (12)	0.0300 (12)	0.0248 (10)	0.0017 (11)	0.0091 (9)	−0.0002 (9)
C32	0.0264 (11)	0.0277 (11)	0.0233 (9)	−0.0010 (9)	0.0018 (8)	0.0022 (8)
C33	0.0302 (11)	0.0260 (11)	0.0238 (10)	0.0026 (9)	−0.0011 (9)	0.0026 (9)
C34	0.0304 (12)	0.0271 (12)	0.0307 (11)	0.0062 (9)	−0.0043 (9)	−0.0030 (9)
C35	0.0373 (12)	0.0212 (11)	0.0422 (13)	0.0002 (10)	−0.0084 (10)	0.0001 (10)
C36	0.0327 (12)	0.0256 (12)	0.0488 (13)	−0.0056 (9)	−0.0014 (11)	0.0050 (11)
C37	0.0251 (11)	0.0295 (12)	0.0342 (11)	0.0005 (9)	0.0020 (9)	0.0031 (10)
C38	0.0234 (10)	0.0217 (10)	0.0216 (9)	0.0006 (8)	−0.0041 (8)	−0.0004 (8)
C39	0.0251 (10)	0.0239 (10)	0.0180 (9)	−0.0004 (8)	0.0021 (8)	0.0001 (8)
C40	0.0306 (11)	0.0215 (11)	0.0305 (10)	−0.0008 (8)	−0.0020 (9)	−0.0001 (9)
C41	0.0260 (11)	0.0343 (12)	0.0344 (11)	0.0020 (10)	−0.0017 (9)	−0.0033 (9)
C42	0.0324 (12)	0.0362 (13)	0.0288 (11)	−0.0099 (10)	0.0028 (9)	−0.0061 (9)
C43	0.0435 (12)	0.0238 (11)	0.0285 (11)	−0.0071 (10)	0.0022 (10)	0.0001 (9)
C44	0.0312 (11)	0.0231 (10)	0.0252 (10)	−0.0010 (9)	0.0005 (8)	0.0024 (8)
C45	0.0299 (11)	0.0189 (10)	0.0217 (9)	−0.0011 (9)	0.0025 (8)	−0.0011 (8)
C46	0.0294 (11)	0.0398 (13)	0.0257 (10)	0.0010 (11)	−0.0010 (8)	−0.0010 (10)
C47	0.0425 (14)	0.0489 (15)	0.0225 (10)	−0.0009 (12)	−0.0034 (9)	−0.0010 (10)
C48	0.0449 (14)	0.0355 (13)	0.0232 (10)	−0.0007 (11)	0.0102 (9)	−0.0028 (9)
C49	0.0320 (11)	0.0284 (12)	0.0331 (11)	−0.0021 (10)	0.0054 (9)	−0.0012 (10)
C50	0.0288 (11)	0.0249 (10)	0.0255 (9)	−0.0017 (9)	−0.0015 (8)	−0.0001 (9)

### Geometric parameters (Å, °)

**Table:**

Ni1—C1	1.9020 (19)	C23—H23A	0.9500
Ni1—P2	2.2305 (9)	C24—C25	1.391 (3)
Ni1—Cl1	2.2327 (8)	C24—H24A	0.9500
Ni1—P1	2.2426 (8)	C25—C26	1.389 (3)
P1—C39	1.827 (2)	C25—H25A	0.9500
P1—C45	1.8381 (19)	C26—H26A	0.9500
P1—C38	1.843 (2)	C27—C28	1.389 (3)
P2—C21	1.829 (2)	C27—C32	1.400 (3)
P2—C15	1.829 (2)	C28—C29	1.396 (3)
P2—C27	1.850 (2)	C28—H28A	0.9500
C1—C2	1.371 (3)	C29—C30	1.378 (3)
C1—C14	1.448 (3)	C29—H29A	0.9500
C2—C3	1.444 (3)	C30—C31	1.383 (3)
C2—H2A	0.9500	C30—H30A	0.9500
C3—C4	1.412 (3)	C31—C32	1.390 (3)
C3—C8	1.414 (3)	C31—H31A	0.9500
C4—C5	1.364 (3)	C32—H32A	0.9500
C4—H4A	0.9500	C33—C34	1.388 (3)
C5—C6	1.407 (4)	C33—C38	1.398 (3)
C5—H5A	0.9500	C33—H33A	0.9500
C6—C7	1.358 (4)	C34—C35	1.386 (3)
C6—H6A	0.9500	C34—H34A	0.9500
C7—C8	1.420 (3)	C35—C36	1.385 (3)
C7—H7A	0.9500	C35—H35A	0.9500
C8—C9	1.450 (3)	C36—C37	1.394 (3)
C9—C10	1.400 (3)	C36—H36A	0.9500
C9—C14	1.433 (3)	C37—C38	1.398 (3)
C10—C11	1.375 (3)	C37—H37A	0.9500
C10—H10A	0.9500	C39—C40	1.392 (3)
C11—C12	1.386 (3)	C39—C44	1.403 (3)
C11—H11A	0.9500	C40—C41	1.387 (3)
C12—C13	1.393 (3)	C40—H40A	0.9500
C12—H12A	0.9500	C41—C42	1.387 (3)
C13—C14	1.403 (3)	C41—H41A	0.9500
C13—H13A	0.9500	C42—C43	1.382 (3)
C15—C16	1.389 (3)	C42—H42A	0.9500
C15—C20	1.400 (3)	C43—C44	1.384 (3)
C16—C17	1.390 (3)	C43—H43A	0.9500
C16—H16A	0.9500	C44—H44A	0.9500
C17—C18	1.387 (4)	C45—C50	1.387 (3)
C17—H17A	0.9500	C45—C46	1.397 (3)
C18—C19	1.375 (4)	C46—C47	1.398 (3)
C18—H18A	0.9500	C46—H46A	0.9500
C19—C20	1.381 (3)	C47—C48	1.373 (3)
C19—H19A	0.9500	C47—H47A	0.9500
C20—H20A	0.9500	C48—C49	1.392 (3)
C21—C22	1.397 (3)	C48—H48A	0.9500
C21—C26	1.401 (3)	C49—C50	1.391 (3)
C22—C23	1.389 (3)	C49—H49A	0.9500
C22—H22A	0.9500	C50—H50A	0.9500
C23—C24	1.398 (3)		
			
C1—Ni1—P2	85.99 (6)	C22—C23—C24	119.7 (2)
C1—Ni1—Cl1	171.79 (6)	C22—C23—H23A	120.2
P2—Ni1—Cl1	93.06 (4)	C24—C23—H23A	120.2
C1—Ni1—P1	87.33 (6)	C25—C24—C23	120.1 (2)
P2—Ni1—P1	171.26 (2)	C25—C24—H24A	120.0
Cl1—Ni1—P1	92.74 (3)	C23—C24—H24A	120.0
C39—P1—C45	103.80 (9)	C26—C25—C24	119.9 (2)
C39—P1—C38	105.14 (9)	C26—C25—H25A	120.1
C45—P1—C38	101.05 (9)	C24—C25—H25A	120.1
C39—P1—Ni1	108.74 (6)	C25—C26—C21	120.70 (19)
C45—P1—Ni1	116.83 (7)	C25—C26—H26A	119.7
C38—P1—Ni1	119.63 (6)	C21—C26—H26A	119.7
C21—P2—C15	105.43 (10)	C28—C27—C32	118.18 (18)
C21—P2—C27	103.49 (9)	C28—C27—P2	120.03 (15)
C15—P2—C27	100.72 (9)	C32—C27—P2	121.77 (15)
C21—P2—Ni1	107.71 (7)	C27—C28—C29	120.5 (2)
C15—P2—Ni1	117.71 (7)	C27—C28—H28A	119.8
C27—P2—Ni1	120.11 (7)	C29—C28—H28A	119.8
C2—C1—C14	118.56 (18)	C30—C29—C28	120.9 (2)
C2—C1—Ni1	118.58 (15)	C30—C29—H29A	119.6
C14—C1—Ni1	122.85 (15)	C28—C29—H29A	119.6
C1—C2—C3	121.7 (2)	C29—C30—C31	119.21 (19)
C1—C2—H2A	119.1	C29—C30—H30A	120.4
C3—C2—H2A	119.1	C31—C30—H30A	120.4
C4—C3—C8	119.2 (2)	C30—C31—C32	120.4 (2)
C4—C3—C2	120.7 (2)	C30—C31—H31A	119.8
C8—C3—C2	120.11 (19)	C32—C31—H31A	119.8
C5—C4—C3	121.2 (2)	C31—C32—C27	120.8 (2)
C5—C4—H4A	119.4	C31—C32—H32A	119.6
C3—C4—H4A	119.4	C27—C32—H32A	119.6
C4—C5—C6	120.0 (2)	C34—C33—C38	121.0 (2)
C4—C5—H5A	120.0	C34—C33—H33A	119.5
C6—C5—H5A	120.0	C38—C33—H33A	119.5
C7—C6—C5	120.1 (2)	C35—C34—C33	120.3 (2)
C7—C6—H6A	119.9	C35—C34—H34A	119.8
C5—C6—H6A	119.9	C33—C34—H34A	119.8
C6—C7—C8	121.6 (3)	C36—C35—C34	119.7 (2)
C6—C7—H7A	119.2	C36—C35—H35A	120.2
C8—C7—H7A	119.2	C34—C35—H35A	120.2
C3—C8—C7	118.1 (2)	C35—C36—C37	120.1 (2)
C3—C8—C9	119.78 (19)	C35—C36—H36A	120.0
C7—C8—C9	122.1 (2)	C37—C36—H36A	120.0
C10—C9—C14	118.7 (2)	C36—C37—C38	120.9 (2)
C10—C9—C8	123.4 (2)	C36—C37—H37A	119.5
C14—C9—C8	117.90 (19)	C38—C37—H37A	119.5
C11—C10—C9	121.6 (2)	C33—C38—C37	117.99 (19)
C11—C10—H10A	119.2	C33—C38—P1	120.79 (15)
C9—C10—H10A	119.2	C37—C38—P1	121.17 (15)
C10—C11—C12	120.2 (2)	C40—C39—C44	119.01 (18)
C10—C11—H11A	119.9	C40—C39—P1	122.85 (15)
C12—C11—H11A	119.9	C44—C39—P1	118.10 (15)
C11—C12—C13	120.1 (2)	C41—C40—C39	119.98 (19)
C11—C12—H12A	120.0	C41—C40—H40A	120.0
C13—C12—H12A	120.0	C39—C40—H40A	120.0
C12—C13—C14	121.0 (2)	C42—C41—C40	120.4 (2)
C12—C13—H13A	119.5	C42—C41—H41A	119.8
C14—C13—H13A	119.5	C40—C41—H41A	119.8
C13—C14—C9	118.52 (19)	C43—C42—C41	120.3 (2)
C13—C14—C1	119.65 (18)	C43—C42—H42A	119.9
C9—C14—C1	121.82 (18)	C41—C42—H42A	119.9
C16—C15—C20	119.42 (19)	C42—C43—C44	119.61 (19)
C16—C15—P2	118.96 (16)	C42—C43—H43A	120.2
C20—C15—P2	121.39 (16)	C44—C43—H43A	120.2
C15—C16—C17	119.7 (2)	C43—C44—C39	120.7 (2)
C15—C16—H16A	120.2	C43—C44—H44A	119.6
C17—C16—H16A	120.2	C39—C44—H44A	119.6
C18—C17—C16	120.3 (2)	C50—C45—C46	118.78 (17)
C18—C17—H17A	119.8	C50—C45—P1	118.76 (15)
C16—C17—H17A	119.8	C46—C45—P1	122.17 (15)
C19—C18—C17	120.2 (2)	C45—C46—C47	120.9 (2)
C19—C18—H18A	119.9	C45—C46—H46A	119.6
C17—C18—H18A	119.9	C47—C46—H46A	119.6
C18—C19—C20	120.1 (2)	C48—C47—C46	119.60 (19)
C18—C19—H19A	120.0	C48—C47—H47A	120.2
C20—C19—H19A	120.0	C46—C47—H47A	120.2
C19—C20—C15	120.3 (2)	C47—C48—C49	120.08 (18)
C19—C20—H20A	119.8	C47—C48—H48A	120.0
C15—C20—H20A	119.8	C49—C48—H48A	120.0
C22—C21—C26	118.82 (19)	C50—C49—C48	120.31 (19)
C22—C21—P2	118.61 (15)	C50—C49—H49A	119.8
C26—C21—P2	122.54 (15)	C48—C49—H49A	119.8
C23—C22—C21	120.79 (19)	C45—C50—C49	120.32 (19)
C23—C22—H22A	119.6	C45—C50—H50A	119.8
C21—C22—H22A	119.6	C49—C50—H50A	119.8
			
C1—Ni1—P1—C39	84.48 (9)	P2—C15—C20—C19	174.90 (16)
P2—Ni1—P1—C39	44.27 (16)	C15—P2—C21—C22	96.93 (17)
Cl1—Ni1—P1—C39	−87.30 (7)	C27—P2—C21—C22	−157.73 (16)
C1—Ni1—P1—C45	−158.52 (9)	Ni1—P2—C21—C22	−29.55 (17)
P2—Ni1—P1—C45	161.27 (15)	C15—P2—C21—C26	−85.05 (18)
Cl1—Ni1—P1—C45	29.69 (8)	C27—P2—C21—C26	20.30 (19)
C1—Ni1—P1—C38	−36.20 (10)	Ni1—P2—C21—C26	148.47 (16)
P2—Ni1—P1—C38	−76.41 (16)	C26—C21—C22—C23	−1.1 (3)
Cl1—Ni1—P1—C38	152.02 (8)	P2—C21—C22—C23	176.97 (16)
C1—Ni1—P2—C21	−86.28 (9)	C21—C22—C23—C24	−0.5 (3)
Cl1—Ni1—P2—C21	85.55 (7)	C22—C23—C24—C25	1.7 (3)
P1—Ni1—P2—C21	−46.01 (17)	C23—C24—C25—C26	−1.1 (3)
C1—Ni1—P2—C15	154.82 (10)	C24—C25—C26—C21	−0.6 (3)
Cl1—Ni1—P2—C15	−33.35 (8)	C22—C21—C26—C25	1.7 (3)
P1—Ni1—P2—C15	−164.90 (15)	P2—C21—C26—C25	−176.32 (16)
C1—Ni1—P2—C27	31.63 (10)	C21—P2—C27—C28	−136.92 (17)
Cl1—Ni1—P2—C27	−156.53 (8)	C15—P2—C27—C28	−28.02 (19)
P1—Ni1—P2—C27	71.91 (17)	Ni1—P2—C27—C28	103.04 (16)
P2—Ni1—C1—C2	88.87 (15)	C21—P2—C27—C32	45.10 (18)
Cl1—Ni1—C1—C2	5.2 (5)	C15—P2—C27—C32	154.00 (17)
P1—Ni1—C1—C2	−85.49 (15)	Ni1—P2—C27—C32	−74.95 (17)
P2—Ni1—C1—C14	−92.31 (15)	C32—C27—C28—C29	0.1 (3)
Cl1—Ni1—C1—C14	−176.0 (3)	P2—C27—C28—C29	−177.97 (16)
P1—Ni1—C1—C14	93.33 (15)	C27—C28—C29—C30	−0.8 (3)
C14—C1—C2—C3	−2.4 (3)	C28—C29—C30—C31	0.8 (3)
Ni1—C1—C2—C3	176.45 (14)	C29—C30—C31—C32	−0.1 (3)
C1—C2—C3—C4	−177.19 (18)	C30—C31—C32—C27	−0.6 (3)
C1—C2—C3—C8	0.1 (3)	C28—C27—C32—C31	0.6 (3)
C8—C3—C4—C5	−1.7 (3)	P2—C27—C32—C31	178.65 (15)
C2—C3—C4—C5	175.6 (2)	C38—C33—C34—C35	−0.2 (3)
C3—C4—C5—C6	0.7 (4)	C33—C34—C35—C36	0.0 (3)
C4—C5—C6—C7	0.5 (4)	C34—C35—C36—C37	−0.6 (3)
C5—C6—C7—C8	−0.8 (4)	C35—C36—C37—C38	1.3 (3)
C4—C3—C8—C7	1.4 (3)	C34—C33—C38—C37	0.9 (3)
C2—C3—C8—C7	−175.90 (19)	C34—C33—C38—P1	−176.66 (16)
C4—C3—C8—C9	179.86 (18)	C36—C37—C38—C33	−1.5 (3)
C2—C3—C8—C9	2.5 (3)	C36—C37—C38—P1	176.07 (17)
C6—C7—C8—C3	−0.2 (3)	C39—P1—C38—C33	−51.45 (17)
C6—C7—C8—C9	−178.6 (2)	C45—P1—C38—C33	−159.19 (16)
C3—C8—C9—C10	177.74 (19)	Ni1—P1—C38—C33	71.01 (17)
C7—C8—C9—C10	−3.9 (3)	C39—P1—C38—C37	131.07 (17)
C3—C8—C9—C14	−2.7 (3)	C45—P1—C38—C37	23.33 (18)
C7—C8—C9—C14	175.66 (19)	Ni1—P1—C38—C37	−106.46 (16)
C14—C9—C10—C11	0.3 (3)	C45—P1—C39—C40	89.94 (17)
C8—C9—C10—C11	179.84 (19)	C38—P1—C39—C40	−15.78 (18)
C9—C10—C11—C12	−0.6 (3)	Ni1—P1—C39—C40	−145.03 (15)
C10—C11—C12—C13	0.5 (3)	C45—P1—C39—C44	−87.64 (16)
C11—C12—C13—C14	−0.2 (3)	C38—P1—C39—C44	166.64 (15)
C12—C13—C14—C9	−0.1 (3)	Ni1—P1—C39—C44	37.40 (16)
C12—C13—C14—C1	−179.99 (18)	C44—C39—C40—C41	−0.8 (3)
C10—C9—C14—C13	0.0 (3)	P1—C39—C40—C41	−178.37 (15)
C8—C9—C14—C13	−179.55 (17)	C39—C40—C41—C42	−1.0 (3)
C10—C9—C14—C1	179.97 (18)	C40—C41—C42—C43	1.7 (3)
C8—C9—C14—C1	0.4 (3)	C41—C42—C43—C44	−0.6 (3)
C2—C1—C14—C13	−177.90 (18)	C42—C43—C44—C39	−1.3 (3)
Ni1—C1—C14—C13	3.3 (2)	C40—C39—C44—C43	2.0 (3)
C2—C1—C14—C9	2.2 (3)	P1—C39—C44—C43	179.63 (15)
Ni1—C1—C14—C9	−176.64 (14)	C39—P1—C45—C50	157.71 (17)
C21—P2—C15—C16	−151.15 (17)	C38—P1—C45—C50	−93.51 (18)
C27—P2—C15—C16	101.48 (18)	Ni1—P1—C45—C50	38.04 (19)
Ni1—P2—C15—C16	−31.05 (19)	C39—P1—C45—C46	−28.5 (2)
C21—P2—C15—C20	34.37 (18)	C38—P1—C45—C46	80.25 (19)
C27—P2—C15—C20	−73.00 (18)	Ni1—P1—C45—C46	−148.20 (16)
Ni1—P2—C15—C20	154.47 (14)	C50—C45—C46—C47	0.1 (3)
C20—C15—C16—C17	−0.7 (3)	P1—C45—C46—C47	−173.63 (19)
P2—C15—C16—C17	−175.27 (19)	C45—C46—C47—C48	1.0 (4)
C15—C16—C17—C18	0.1 (4)	C46—C47—C48—C49	−1.4 (4)
C16—C17—C18—C19	0.7 (4)	C47—C48—C49—C50	0.5 (4)
C17—C18—C19—C20	−0.9 (4)	C46—C45—C50—C49	−1.0 (3)
C18—C19—C20—C15	0.4 (3)	P1—C45—C50—C49	173.00 (16)
C16—C15—C20—C19	0.4 (3)	C48—C49—C50—C45	0.7 (3)
